# *Moringa oleifera* as an Anti-Cancer Agent against Breast and Colorectal Cancer Cell Lines

**DOI:** 10.1371/journal.pone.0135814

**Published:** 2015-08-19

**Authors:** Abdulrahman Khazim Al-Asmari, Sulaiman Mansour Albalawi, Md Tanwir Athar, Abdul Quaiyoom Khan, Hamoud Al-Shahrani, Mozaffarul Islam

**Affiliations:** Research Center, Prince Sultan Military Medical City, Riyadh, 11159, Kingdom of Saudi Arabia; University of Alabama at Birmingham, UNITED STATES

## Abstract

In this study we investigated the anti-cancer effect of *Moringa oleifera* leaves, bark and seed extracts. When tested against MDA-MB-231 and HCT-8 cancer cell lines, the extracts of leaves and bark showed remarkable anti-cancer properties while surprisingly, seed extracts exhibited hardly any such properties. Cell survival was significantly low in both cells lines when treated with leaves and bark extracts. Furthermore, a striking reduction (about 70–90%) in colony formation as well as cell motility was observed upon treatment with leaves and bark. Additionally, apoptosis assay performed on these treated breast and colorectal cancer lines showed a remarkable increase in the number of apoptotic cells; with a 7 fold increase in MD-MB-231 to an increase of several fold in colorectal cancer cell lines. However, no significant apoptotic cells were detected upon seeds extract treatment. Moreover, the cell cycle distribution showed a G2/M enrichment (about 2–3 fold) indicating that these extracts effectively arrest the cell progression at the G2/M phase. The GC-MS analyses of these extracts revealed numerous known anti-cancer compounds, namely eugenol, isopropyl isothiocynate, D-allose, and hexadeconoic acid ethyl ester, all of which possess long chain hydrocarbons, sugar moiety and an aromatic ring. This suggests that the anti-cancer properties of *Moringa oleifera* could be attributed to the bioactive compounds present in the extracts from this plant. This is a novel study because no report has yet been cited on the effectiveness of Moringa extracts obtained in the locally grown environment as an anti-cancer agent against breast and colorectal cancers. Our study is the first of its kind to evaluate the anti-malignant properties of Moringa not only in leaves but also in bark. These findings suggest that both the leaf and bark extracts of Moringa collected from the Saudi Arabian region possess anti-cancer activity that can be used to develop new drugs for treatment of breast and colorectal cancers.

## Introduction

*Moringa oleifera* L (MO) (Family: Moringaceae) is a perennial angiosperm plants, which includes several other species [1, 2]. It is a native of the Himalayan region that is widely cultivated throughout tropical and sub-tropical countries of the world including Saudi Arabia. [3, 4]. The plant has numerous medicinal applications and is used as a traditional medicine for the treatment of various illnesses such as skin diseases, respiratory distress, ear and dental infections, hypertension, diabetes, anemia, and cancer [5–9]. Additionally, the pharmacological importance of the leaves extract containing bio-active compounds are well described by Leone *et al* (2015) [10].

In this study we focused upon the effect of *Moringa oleifera* extracts from leaves (MOL), bark (MOB), and seeds (MOS) to observe its efficacy as an anti-cancer agent on breast and colorectal cancers. To elucidate the effectiveness of these extracts we analyzed cell motility and clonogenic survival assays to assess the phenotypic changes in MDA-MB-231(breast) and HCT-8 (colorectal) cancer cell lines. To elaborate our study further, we also analyzed the cell survival, apoptosis, and cell cycle progression of these two lines after challenging them with the extracts of MO as stated above. The rationale behind selecting these lines are; easy access to availability and more importantly, prevalence of these cancers in a major population of the Kingdom of Saudi Arabia.

Colorectal cancer is the third most lethal cancer worldwide. Both genders, male and female are equally affected by this deadly disease. In the past year about 140,000 people were diagnosed with colon cancer in the United States and the estimated survival is 50% or less[11]. Furthermore, the Saudi Cancer Registry has reported a sharp increase in colorectal cancer in the kingdom [12]. Similarly, breast cancer among women is also a deadly cancer worldwide [13]. A study conducted between 2001–2008 reports a significant rise in breast cancer among young women in Saudi Arabia. Noticeably, the incidence is more prominent in the eastern province of the kingdom consequently the women in these areas are more vulnerable to this disease [14].

The important characteristic features of cancer cells include the ability to proliferate, invade through the extra cellular matrix and migrate to other body parts to form secondary tumors. The migration of cancerous cells is dependent on the tumor micro environment from where they get nourishment and support by forming new-vasculature (a process called angiogenesis) and allowing them to spread [15]. It is a challenging task for Oncologists and Medical Scientists to devise the best treatment regimen that kills the maximum number of cancer cells with minimal side effect rendering maximum benefits to the cancer patients.

As reported earlier, about 74% of the known anti-cancer medicines are derived from various plant species [16, 17]. Indeed, there are many household dietary products exhibiting anti-cancer potential with minimal side effect that are currently under clinical trials for cancer treatment [18, 19]. Among these, two important household dietary products that are very common among South Asian communities are Curcumin and Lycopene. Curcumin is a polyphenolic compound isolated from turmeric and this product exhibits anti-microbial, immunomodulatory, and potential cancer chemo preventive efficacy [20, 21].

Lycopene is a carotenoid compound abundant in tomatoes and also present in tomato products [22]. As reported earlier lycopene and its derivative exhibit anti-cancer properties. In addition to this the product can also be used in the treatment of cardiovascular diseases [23]. The anti-cancer properties of curcumin are well studied. The molecular mechanism, by which curcumin acts is by inhibiting MAP kinase activity, negatively interfering with JAK/STAT signaling pathways, and inhibiting the expression of several transcription factors including NF-Kβ and STAT [24]. The expression of apoptotic proteins such as caspases and the anti-apoptotic protein Bcl-2 were also reported to be modulated by curcumin [25, 26]. On the other hand, the anti-cancer mechanism of lycopene acts by inhibiting the PI3K/AKT signaling pathways as well as inducing apoptosis in the cancerous cells [27, 28].

In this study we have shown the remarkable effects of Moringa leaves and bark on MDA-MB-231 and HCT-8 cancer cell lines. Moringa is a common vegetable used by inhabitants of tropical and sub-tropical countries. The extracts of leaves and bark tested in our current study induced a significant level of apoptosis as well as G2/M enrichment in breast and colorectal cancer cell lines. In addition, a remarkable change in the normal phenotypic properties of the cells was also observed. However, surprisingly, we did not observe any significant changes in cell lines exposed to Moringa seed’s extract. Furthermore, when GC-MS analyses of these extracts were performed, we observed considerable amounts of bioactive components present in the extracts of leaves and bark, which we have describe elaborately in the results section. The effective role of these compounds present in the extracts as an anti-cancer agent is well documented [29–32]. Therefore, the aim of this study was to evaluate the anti-cancer potential of *Moringa oleifera* grown in the kingdom of Saudi Arabia, against breast and colorectal cancers. The importance of this study is that this locally grown plant has not been previously tested as an anti-cancer agent. Hence, the novelty of our research is that we have tested not only leaf extracts, but also the bark and the seed extracts against two cancer cell lines, which has not been studied in the past.

## Materials and Methods

### Collection of plant materials

Leaves (L), bark (B), and seeds (S) of *Moringa oleifera* were collected from the city of Tabuk, Kingdom of Saudi Arabia. Geographically the latitude of the city is 28^°^ 22`60N and longitude is 36^°^ 34`60E on degree minutes second (DMS) unit. Due to the ease of cultivation and abundance, it does not fall under the endangered plant species and hence safe to use for research purpose. (http://faculty.ksu.edu.sa/74413/Pages/Endangeredplants.aspx). Additionally, the parts of the plant used in this study are the gift of one of the co-authors (SMA) of this manuscript obtained from his garden plants. The study was performed in the research center, Prince Sultan Military Medical City, Riyadh.

### Preparation of the extracts

The extractions were performed using Soxhlet apparatus. About 60g of dried leaves, bark, and seeds were grinded separately into coarse powder. Each of the coarsely powdered specimens was taken in round bottom flask and 600 ml of ethanol was added. The extraction was continued for 6–8 h until all the soluble constituents dissolved in the solvent. The soluble extracts were filtered and evaporated in rotary evaporator (Buchi, Switzerland; temp: 50°C; pressure 175 mbar) to yield semi solid masses. Extracts thus obtained, were collected and stored at 4°C until further use. For this study 250mg of extracts were dissolved in 1.0 ml of ethanol and filtered through a 0.22μM filter. The sterile extracts were always used in a cell culture hood under aseptic conditions.

#### Gas chromatography- mass spectrometry (GC-MS) analyses

GC-MS analysis of the extracts were carried out in a GC system (Agilent 7890A series, USA) equipped with split/splitless injector and auto-sampler attached to an apolar 5-MS (5% phenyl polymethyl siloxane) capillary column (Agilent 19091S-43; 30 m×0.25 mm i.d. and 0.25-μm film thickness) and fitted to Mass Detector (Agilent 5975C series, USA). The flow rate of the carrier gas, helium (He) was set to be at 1 ml.min^−1^ in split less mode. The injector temperature was adjusted at 250°C, while the detector temperature was fixed to 280°C. The column temperature was kept at 70°C for 1 min followed by linear programming to raise the temperature from 70° to 200°C (at 8°C min^−1^ with 2 min hold time), and 200°C to 250°C (at 10°C min^−1^ with 2 min hold time). The transfer line was heated at 280°C. Total run time was 27.2 min. Mass spectra were acquired in scan mode (70 eV); in the range of 50 to 550 m/z. Twenty microliter each of the extracts (250 mg/ml stock) were further diluted in 2 ml of methanol. One micro liter of this diluted sample was injected for GC-MS analyses.

### Identification of compounds

Interpretation of mass spectra was conducted using the database of the National Institute of Standards and Technology (NIST, USA). The database caters for more than 62,000 patterns of known compounds. The spectrum of the extracts was matched with the spectrum of the known components stored in the NIST library.

### Cell culture

Cancer cell lines namely HCT-8 (derived from the ileocecal adenocarcinoma of a 67 year old male [33]; and MDA-MB-231 (obtained from breast mammary glands) [34] were kindly supplied by the cancer research facilities, King Saud Bin Abdulaziz Medical City, Riyadh, Kingdom of Saudi Arabia and had been originally procured from the American Type Culture Collection (ATCC), USA. These cell lines were cultured either in RPMI-1640 for HCT cell line or DMEM (Life technologies, USA) for MDA-MB-231 cell line, supplemented with 10% heat inactivated fetal bovine serum (PAA Laboratories, Germany), 2mM L-glutamine, 50μg/mL of penicillin-G, and 50μg/mL of streptomycin sulfate. The culture was maintained as described earlier [35].

### Assays to determine the phenotypic changes

- **Motility assay:** cell motility for MDA-MB-231 and HCT-8 lines was performed in 6 well cell culture plates as described earlier [36]. Cells were allowed to grow either in absence (control) or in presence of MOL, MOB, and MOS (250μg/ml and 500 μg/ml). The extent of the gap filled by the cells was monitored microscopically after 24 hours of treatment.
- ***In vitro* Clonogenic survival assay:** Anchorage dependent colony formation assay was performed according to the standard procedure [37, 38]. The results of the assay were depicted in the form of photographs and the quantitative analysis was shown in the form of bar graphs.

### Cell viability, apoptosis and cell cycle arrest assays

All three cellular parameters were examined on the Muse Cell Analyzer (Millipore, USA).

- ***Cell viability assay*:** the cell viability assay was performed in 24-h pre-treated MDA-MB-231 and HCT-8 cell lines by different concentrations of plant extracts. The assay was performed as per the manufacturer’s protocol. Briefly, 12x10^4^ cells from control and extract treated cells were taken in 200 μl of PBS and mix with 380μl of cell counting solution (Millipore, USA cat # MCH 100102). Contents were gently mixed for a few seconds and immediately read on the machine using specific programming. A histogram and numeric values displayed the number of dead and live cells after treatment.
- ***Apoptosis assay*:** in order to evaluate the extent of early and late apoptosis induced by the plant extracts, 12x10^4^ cells from MDAMB-231 and HCT-8 cell lines were treated for 24 h with different concentrations of leaves, bark and seeds extracts. Thereafter the extent of apoptosis was examined on the Muse cell analyzer. The assay was performed by utilizing the Annexin V and Dead Cell Kit (Millipore, USA cat # MCH 100105). Using designated programming on the cell analyzer, the numbers of live, dead, early and late apoptotic cells were determined. Total apoptosis was calculated by combining the number of cells from late and early apoptosis quadrants of the histograms and is presented in the form of bar graphs.
- ***Cell cycle assay*:** to determine the effect of plant extracts on cell cycle arrest the assay was performed using the cell cycle kit (Millipore, USA cat # MCH100106). At the completion of the plant extracts treatment which was done in the same way as described above, cells were trypsinized, and counted. About 12x 10^4^ cells from control and treated groups were fixed in chilled 75% ethyl alcohol for 3h at -20°C. Next cells were washed once with PBS and incubated with 200μl of assay solution for 30 min in the dark at room temperature. After completion of the incubation period the cells were vortexed gently and read on the cell analyzer. The number of cells at each event namely, G_o_/G1, S and G2/M phases were determined in control and extracts treated cells.

### Statistical calculation

Statistical calculations (Student’s t-test) were performed using Graph Pad Prism 4.0 software. The mean was reported with standard deviation (± SD). Differences were considered to be statistically significant when *p* values were ≤ 0.05.

## Results

### Optimization of GC-MS Method

GC method was optimized by varying the oven temperature. In current gradient oven temperature programming, a good resolution of all the extracts has been seen in a relatively short duration of time. The fragmented ions were separated by the analyzer, according to their mass-to-charge ratio.

#### GC-MS analyses of *Moringa oleifera*’s (MO) leaves, barks and seeds show several carbohydrates/ sugar and long chain fatty acid moieties

**Fig 1:**
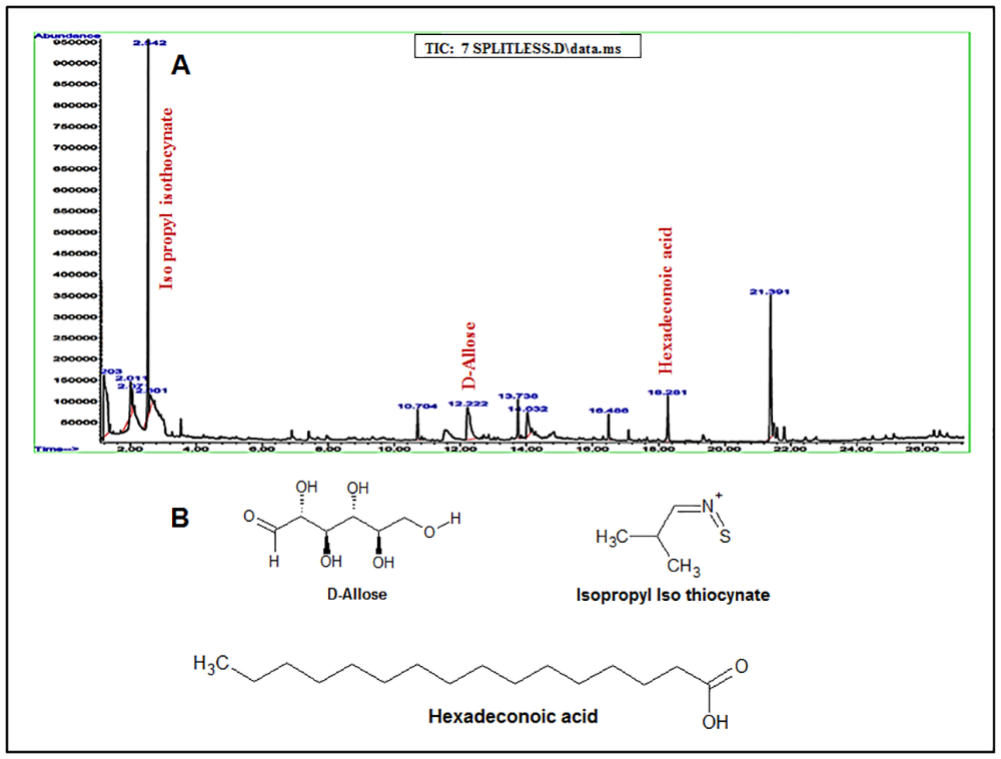
(A) GC-MS analyses of MO leaves. Typical TIC-GC/MS chromatogram of ethanolic extract of Moringa leaf analyzed on GC system (Agilent 7890A series, USA) equipped with an apolar 5-MS (5% phenyl polymethyl siloxane) capillary column (Agilent 19091S-43; 30 m×0.25 mm i.d. and 0.25-μm film thickness) attached with Mass Detector. After comparing with the spectra in the NIST Library, It showed the presence of twelve compounds. To facilitate the interpretation, only the peaks showing bioactive anticancer compounds such as isopropyl thiocynate, D-allose, and hexadeconoic acid are marked. (B) Chemical structures of the active compounds as mentioned above are shown here.

**Fig 2:**
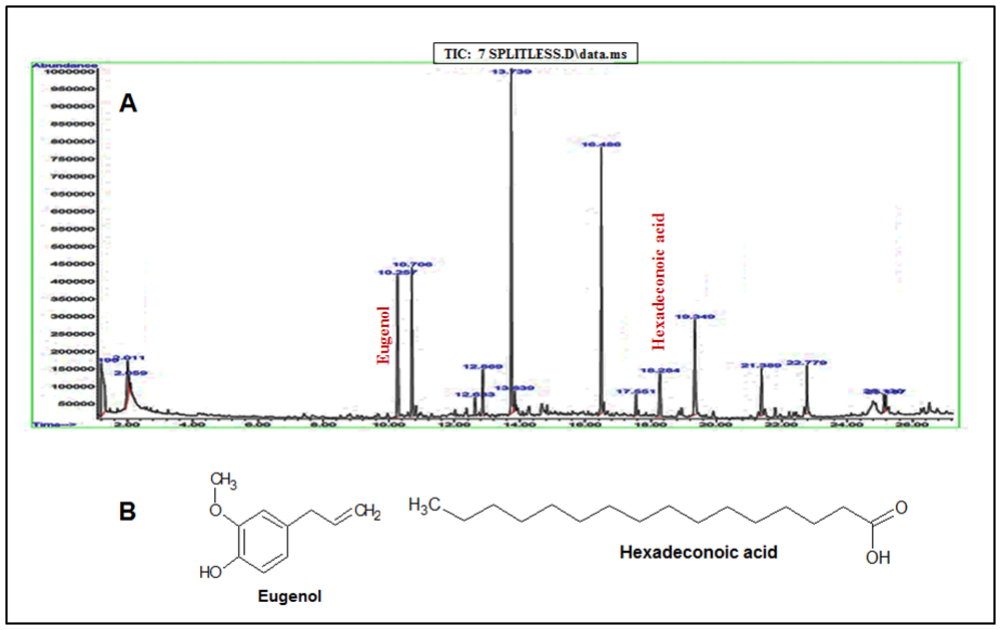
GC-MS analyses of MO bark. (A) Typical TIC-GC/MS chromatogram of Moringa bark analyzed on GC system equipped with an apolar 5-MS capillary column attached with Mass Detector. It showed the presence of seventeen compounds. The peaks showing bioactive anticancer compounds in bark such as eugenol and hexadeconoic acid are marked. (B) Chemical structures of the active compounds as mentioned above are shown here.

The ethanol extracts of the leaves and bark of Moringa showed twelve and seventeen peaks on chromatogram respectively (Figs 1A and 2A). The extracts of leaves mainly comprises of thiocynates, hydrocarbons and fatty acids, while the bark extract chiefly consists of hydrocarbons, phenolics, phthalates, carboxylic acids, and long chain fatty acids. Analyses of seeds revealed either hydrocarbons or long chain fatty acids and its derivative (S1 Fig). Chemically, the composition of leaves, bark and seeds revealed on GC-MS, were isopropyl isothiocyanate, D-allose and cetene present exclusively in the leaves extract; eugenol, dibutyl phthalate, 2- chloropropionic acid and 5-eicosene, present only in the bark extract. The seeds extracts showed the presence of 1-butanamine, 1-dodecene, 2-decenal, 3-tetradecene, 2-tetradecene, 1-octadecene, hexadecanoic acid, 10-octadecenoic acid, and heptadecanoic acid. Some of the compounds and derivatives like octadecene (C18:1 in leaf & C18:1 in bark), tetradecene (C14:5 in leaf & C14:2 in bark), octadeconoic acid (C18:11 in leaf & C18:9 and C18:9 in bark) and palmitic acid has been found in both leaf and bark extracts. Interestingly, we did not detect phenolic compounds and flavonoids using GC-MS techniques. Our findings are in accordance with the previously published work [39]. The molecular structures of some of the bioactive molecules are given in Figs 1B and 2B.

#### Extracts of MO leaves and bark significantly reduced the cell survival of breast and colorectal cancer cell lines

**Fig 3:**
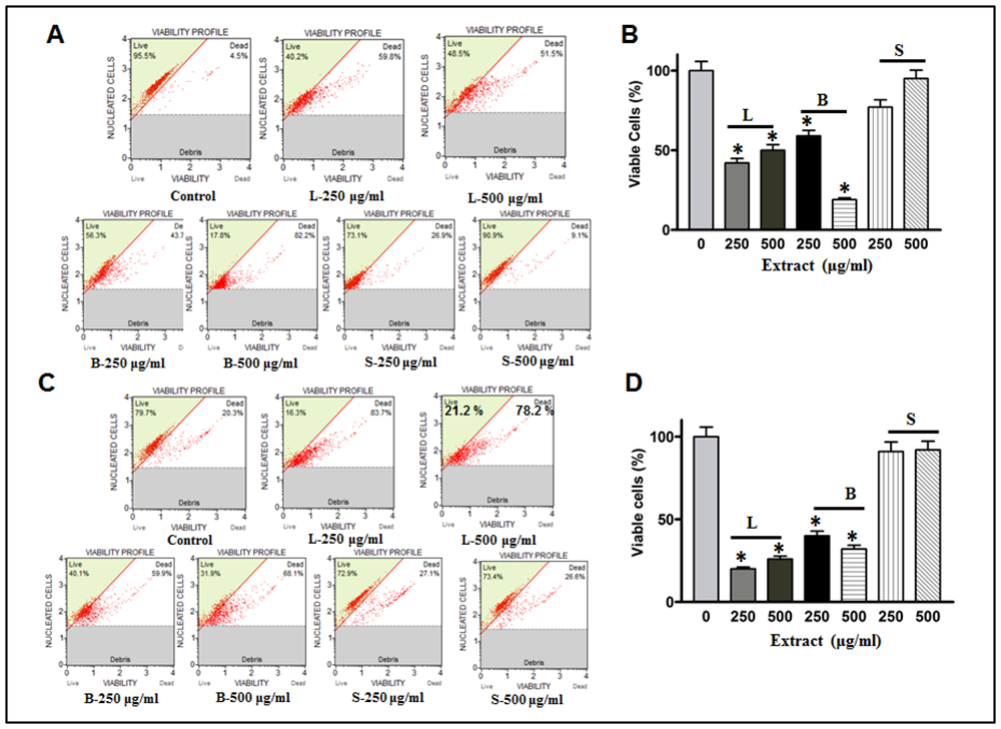
Cell survival assay. (A) MDA-MB-231 (B) HCT-8 cell lines. Cells were treated with different concentrations of plant extracts; (L) leaves; (B) bark and (S) seed. Equal numbers of cells in 20 μl were taken in 380 μl of cell counting solution. Cell viability was analyzed on Muse cell analyzer. Column (B & D) representing the quantitative analysis of the viable cells. A significant number of dead cells were observed upon extract treatment as compared with control. * Statistically significant (*P*≤0.05).

The effect of MOL, MOB, and MOS were tested on MDA-MB-231 and HCT-8 cell survival. The analysis was done on the Muse Cell Analyzer as described in the Materials and Methods section. A significant decrease in cell survival was observed in both MDA-MB-231 and HCT-8 cancer cell lines when treated with the extracts of leaves and bark. Interestingly, we did not observe any remarkable decrease in cell population when these lines were exposed to the seed extract of MO. A bar graph was constructed summarizing the effect of MO extracts on growth of these two cancer cell lines (Fig 3A–3D). The selection of the dosages of these extracts was based on our preliminary studies on cell survival, where we found that a concentration of 250 μg/ml and below did not significantly reducing the cell survival. Furthermore, concentrations over 500 μg/ml become toxic and consequently cell disruptions were very high. Taken together, this study strongly suggests a “switching off” of cancer cell survival mechanisms under the influence of MO extracts. Further studies based on the molecular events and signaling cascade could be pursued in future research. At this point, we elaborated our study to examine an important clinical question concerning how these extracts inhibit cell survival. In pursuant to this query we analyzed the phenotypic changes in the cells. An important observation was that a lower number of cells survived extract treatment compared to the control, suggesting cell death or reduction in cell proliferation. Therefore, we examined the effect of MO extracts on apoptosis and their effect on cell cycle arrest in these cell lines.

#### Functional analysis of MO extracts on MDA-MB-231 and HCT-8 cell lines

**Fig 4:**
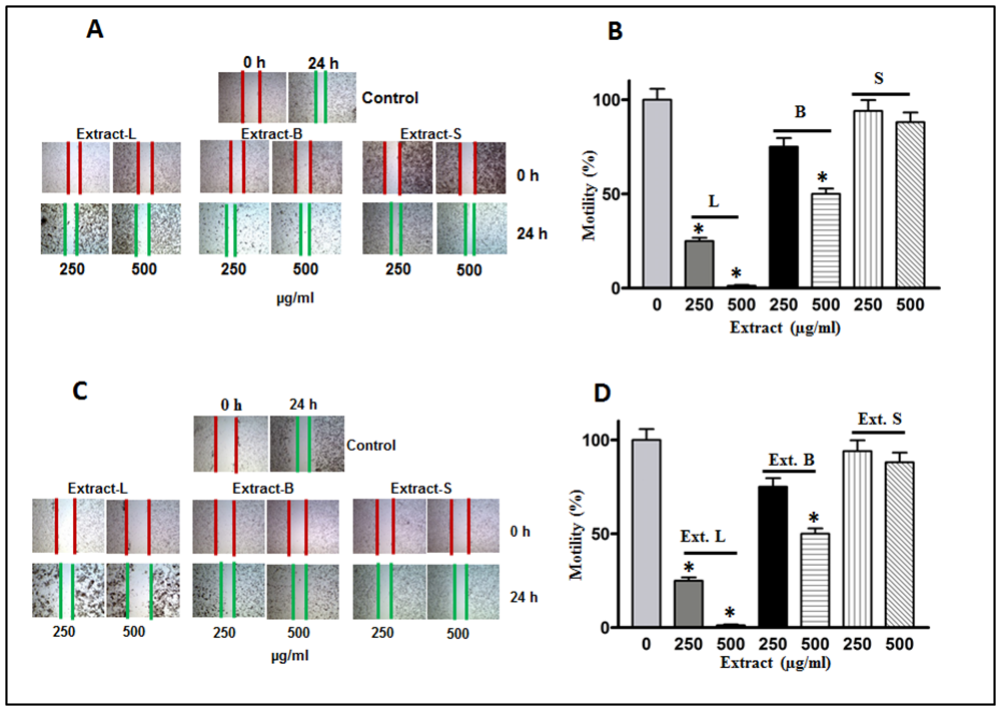
Cell motility assays. (A) MDA-MB-231 and (C) HCT-8 cell lines. Treatment patterns and annotation of the extracts were the same as descried in fig 3. A significant decrease in cell motility was observed upon extracts treatment. Columns of the bar graphs (B&D) showing 80–95% decrease in cell motility. * Statistically significant (*P*≤0.05).

**Fig 5:**
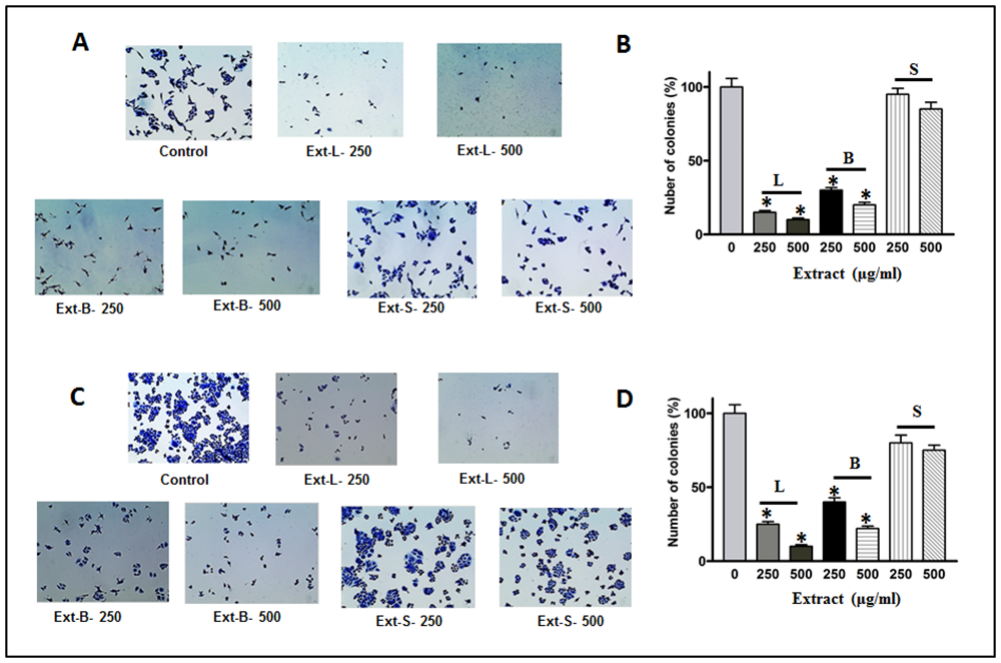
Colony formation assay. Anchorage dependent colony formation assay shows a significant reduction in colony formation in (A) MDA-MB-231 and (C) HCT-8 cell lines treated with different concentration of plant extracts. Quantitative analyses are given in the form of bar graphs for MDA-MB-231(B) and HCT-8 (D) respectively. A dramatic decrease in colony formation is evident from the figure. Statistically significant values were marked with asterisk. (*P*≤0.05).

As discussed above, the extracts of MO (leaves and bark) showed a promising effect on the inhibition of cell survival. Next, we performed a cell motility assay using all three extracts of MO. As expected, our cell motility assay shows a significant decrease in wound closing after 24-h of treatment of cell lines with the extracts when compared with corresponding controls. A noticeable decrease of about 90% in leaves extract and about 50% in bark extract was observed when cells were treated at 500μg/ml concentration in both MDA-MB-231 and HCT-8 cell lines (Fig 4A–4D). However, we did not observe any significant inhibition in cell motility in the cells which were exposed to the seed extract. Furthermore, anchorage independent colony formation is an immensely important parameter in cancer survival and progression. In this study we observed a perceptible decrease in colony formation in MDA-MB-231 and HCT-8 cell lines when treated with different concentrations of leaves and bark extracts respectively (Fig 5A and 5C). The corresponding bar graph shows the quantitative analysis of the colonies formed (Fig 5B and 5D). As shown in the figures, about 80–90% reduction in colony formation was observed in leaves extract treated cells and an almost similar effect was also observed upon bark treatment. As expected, the cells were unresponsive when treated with the seed extracts in this assay also. The bar graphs are the mean of the count of five independent colonies in a high power field and a representative field is shown in the figures. Similarly, for motility assay bar graphs are the average of three independent experiments and a representative of the visual field has been depicted in all the figures. Next, we explored the probable reason for the dramatic reduction of normal function of these lines when treated with the MO extracts. We used two well established parameters to determine the change in cellular architecture of the cell lines used in this study under the treatment conditions as stated above.

#### *Moringa oleifera* leaves and bark induce late apoptosis in MDA-MB-231 and HCT-8 cell lines

**Fig 6:**
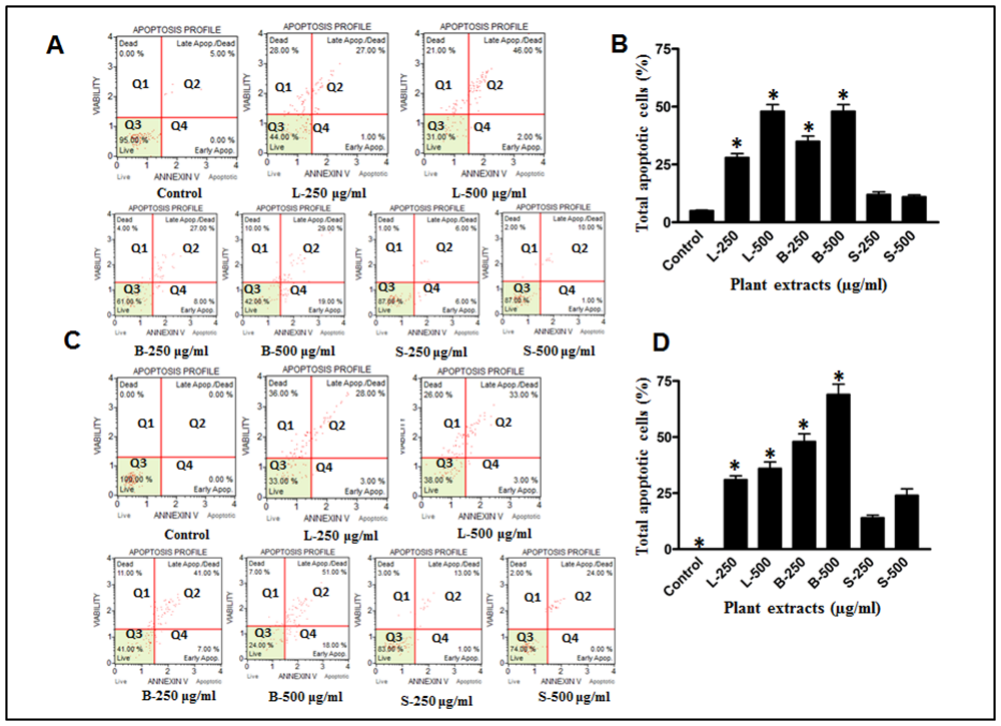
Apoptosis assay. Assessment of apoptosis in (A) MDA-MB-231 and (C) HCT-8 cell lines treated with the extracts of MO leaves (L), bark (B) and seed (S) for 24 h. The cells cultured either in DMEM or in RPMI media were used as control. The apoptotic rates were detected by annexin V-PI dual staining. Q1 quadrant (annexin V−, PI+) represented dead cells; Q2 quadrant (annexin V+, PI+) represented late apoptotic cells; Q4 quadrant (annexin V+, PI–) represented early apoptotic cells; Q3 quadrant (Annexin V−, PI−) represented live cells. The percentage of total apoptotic cells (Q2+Q4) was calculated and shown in the bar graphs MDA-MB-231(B) and HCT-8 (D) cell lines respectively. The extent of apoptosis was significantly high both in leaves and bark treated cell lines. However, few apoptotic cells were also observed only in HCT-8 when treated with the seed extract. * Statistically significant (*P*≤0.05).

Our consistent findings on cell survival and also on phenotypic assays gave an indication that the low rate of cell survival after extract treatments could be due to the induction of apoptosis. In order to assess the degree of apoptosis caused by the MO extracts on breast and colorectal cancer cell lines, an apoptosis assay was performed on the Muse cell analyzer using the Annexin V staining procedure. The extent of apoptosis was increased as a function of extract concentrations. In this study we observed a significantly high population of late apoptotic cells when treated with plant extracts as compared to control. The number of apoptotic cells increase from 27% to 46% in leaves treatment and from 27% to 29% in case of bark treated MDA-MB-231 cell lines, while control shows only 5% late apoptotic cells (Fig 6A and 6C). A similar increase in late apoptotic cells was also observed in HCT-8 cell lines. As expected, extract of seed does not show any noticeable changes in the status of cell lines upon treatment. Statistical analyses of the findings were depicted in the form of bar graphs (Fig 6B and 6D).

#### G2/M phase arrest was evident in MO treated cell lines

**Fig 7:**
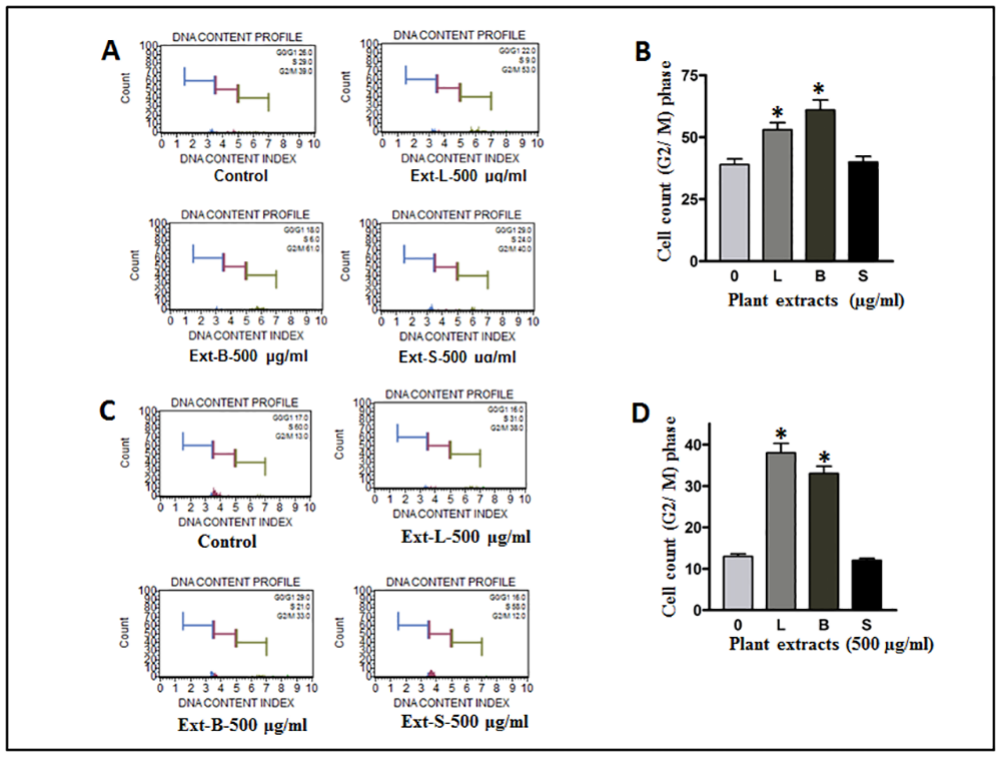
Cell cycle assay. Analysis of cell cycle arrest in plant extract treated (A) MDA-MB-231 and (C) HCT-8 cell lines. Cells were treated with 500μg/ml extracts of leaves (L) Bark (B) and seeds(S). Statistical analyses of the findings were shown in the form of bar graphs for MDA-MB-231(B) and HCT-8 (D) cell lines respectively. A significant G2/M enrichment was observed in leaves (L) and bark (B) treated cell lines. * Statistically significant (*P*≤0.05).

To elucidate the mechanism of reduced cell survival and cell movement, the effect of MO extracts was investigated on cell cycle progression. MDA-MB-231 and HCT-8 cells were treated with 500μg/ml concentration of leaves, bark and seed extracts for 24-h. At the termination of the treatment time (24-h), the cell cycle distribution analysis was performed on the Muse cell analyzer. As shown in fig 7A and 7B, G2/M enrichment was significantly higher in MDA-MB-231 cell lines. It varied from 29% (control) to 53% in leaves and 61% in bark respectively upon treatment. Additionally, an increase in G2/M accumulation from 13% (control) to 38% and 33% in leaves and bark treatments respectively were also observed in HCT-8 cell line (Fig 7C and 7D). As noted above, extracts of MO seed did not exhibit any role in cell cycle arrest either in MDA-MB-231 or in HCT-8 cell lines.

## Discussion

*Moringa oleifera*, a common vegetable plant in many Asian and South East Asian countries possesses numerous compounds with excellent health benefits including anti-oxidant and anti-cancer properties [40]. The plant exhibits anti-cancer potential by interfering with the signal transduction cascade that promotes cancer cell proliferation and progression [9]. The inhibition of cancer cell proliferation is mainly due to the presence of eugenol, a phenolic natural compound which targets E2F1/survivin in cancer cells [29], D-allose [30], isopropyl isothiocynate [31] etc. Keeping in view the anti-cancer properties of MO we hypothesized it may be an effective treatment for breast and colorectal cancers using locally grown plants. The distinctiveness of this study is that no one in the past has reported on the anti-cancer properties of MO cultivated in the Kingdom of Saudi Arabia. In this study, we evaluated the anti-cancer properties of MO leaves, bark and seeds extracts against MDA-MB-231 (breast) and HCT-8 (colorectal) cancer cell lines. Our first and foremost aim was to obtain the details of the chemical compounds present in these three components (as mentioned above) of MO. The GC-MS analyses revealed numerous anti-cancer compounds present in the extracts of leaves and bark of MO. Surprisingly; we did not find any significant anti-cancer compound(s) in the seed extracts (S1 Fig). Furthermore, the GC-MS analyses of Moringa extracts (leaves, bark and seeds) showed a number of phyto-constituents in the chromatogram. Most of these constituents possess anticancer activity against cancer cell lines and/or *in vivo* models. There is evidence that D-allose (present in leaves of Moringa) inhibits the growth of cancer cells at G1 phase (G1- cell cycle arrest) through specific thioredoxin interacting protein (TXNIP) induction and subsequent p27kip1 protein stabilization without exerting appreciable effects on normal cells [41]. The organosulphur compound has been shown to have anti-cancer properties in an *in vivo* model [39]. The presence of isothiocynate (organosulphur compound) in MO bark extract can be attributed to its anti-cancer property. Hexadecanoic acid (palmitic acid) present in all parts (leaf, seed and bark) of Moringa has been found to have selective cytotoxicity against human leukemic cells, as well as *in vivo* anti-tumor activity in mice [42]. Previous studies also showed that eugenol (found in bark of Moringa) has a strong anticancer potential against melanoma [43–45], osteosarcoma [46], leukemia [47], gastric cancer [48, 49], skin tumor [50, 51], mast cells [52] and prostate cancer [53]. In summary, the GC-MS-analyses demonstrate that the extracts of Moringa leaves and bark have a number of bioactive anti-cancer constituents which might be responsible for its strong anti-cancer activity against MDA-MB-231 and HCT-8 cancer cell lines. Furthermore, when we tested these compounds against MDA-MB-231 and HCT-8 cell survival, a remarkable decrease in viable cells was detected on the Muse cell analyzer. The effects of the leaves and bark extracts on cell survival were significantly effective within minor variation in the number of viable cells. This variation in the number of viable cells could be due to the differential cellular uptake mechanism in this particular set of experiment. What we found was that the cell survival was significantly reduced in the presence of these extracts as compared with the corresponding control (Fig 3A–3D). The decrease in cell number can be attributed to the chemical compounds found in the extracts as discussed above, which promotes the inhibition of cancer survival protein expression [29]. Next, we focused upon the phenotypic changes in these cell lines when challenged with MO extracts. Cell motility and anchorage independent colony formation assay, which are hall marks of cancer progression and survival revealed dramatic decrease in cell motility and colony formation. Furthermore, the cytotoxic effect of MO could also contribute towards the change in the cellular phenotype in these lines. However, the cell lines exposed to seed extract were unresponsive. These findings signify the role of leaves and bark extracts as anti-cancer agents (Figs 4 and 5). Our results are in good agreement with previous work demonstrating MO leaves possessed anti-cancer properties [8]. In this study, we have extended our strategy by utilizing the extracts not only from leaves as reported earlier, but also the extracts obtained from bark and seeds. Additionally, we examined cell motility under the influences of these extracts in MDA-MB-231 and HCT-8 cell lines. This cell motility step which is a new addition to this field gave additional information about the inhibitory role of MO by reducing the cell motility phenomenon of breast and colorectal cancer cell lines.

Lacroix *et al* (2006) reported the role of eugenol (which is also present in MO bark) in MDA-MB-231 cell apoptosis by increasing Bax protein expression [54]. Following the same mechanism of apoptosis caused by eugenol, Al-Sharif *et al* (2013) reported the possible mechanism of action of eugenol by down regulating the E2F1 protein which shows promising outcomes in breast cancer [29]. Our results are also in accordance with the reports obtained on leaves extract as discussed above. As shown in Fig 6A–6D, a perceptible increase in total numbers of apoptotic cells was detected when the cells from breast and colorectal cancers were treated with leaves and bark extracts of *Moringa oleifera*. The rate of increased apoptosis is gradual and also dose dependent. In conclusion, the increased apoptotic events in the presence of MO extracts could be the manifestation of down regulation of E2F1 and up-regulation of Bax proteins. A study by Berkovich *et al* (2013) showed similar findings in which cell cycle arrest was observed in a pancreatic carcinoma cell line when treated with Moringa leaves extract [55]. In this study, we have shown a significant G2/M enrichment not only in cells treated with leaf extracts, but also in bark extract treated breast and colorectal cancer cell lines (Fig 7A–7D). The overall mechanism of reduced cell viability and active participation in normal cellular activity could be attributed to the “shutting down” of various cancer survival pathways including the NF-Kβ signaling cascade by down regulating the important component p65 of NF-kβ by the compounds present in Moringa extracts [55].

The most widely used and accepted method for obtaining plant extracts is the Soxhlet technique and this operates at considerable amount of heat. Consequently the chances of thermal inactivation of the bio-active compounds cannot be ruled out. Therefore, we should still consider this step of extraction as the limitation of this technique.

The implication of this study opens a new and fertile area of future research to elucidate the molecular mechanism by which signaling events take place after treating cancer cells with MO extracts. There are two probable possibilities which need future investigation. First, a decrease in the expression of PI3K/AKT pathway targeting proteins or second, inhibition of the phosphorylation of focal adhesion kinase (p-FAK), which plays a significant role in cell migration and in cell invasion.

In summary, we show that the MO extracts act as an anti-cancer agent by decreasing cell motility and colony formation in colorectal and breast cancer cell lines. Additionally, low cell survival, high apoptosis and G2/M enrichment was detected upon treatment with the extracts of MO leaves and bark. In conclusion, our findings add to the growing evidence supporting the promising role of MO as an anti-cancer agent and open a new vista for molecular analysis of the action of MO on the predominant signaling mechanisms responsible for cancer development. Therefore, MO extracts may represent a valuable therapeutic tool for use as part of a therapy for the treatment of aggressive breast and colorectal carcinoma.

## Supporting Information

S1 Fig GC-MS analyses of MO seed. (A) Typical TIC-GC/MS chromatogram of Moringa seed analyzed on GC system equipped with an apolar 5-MS capillary column attached with Mass Detector. It showed the presence of fourteen compounds, these are: 1-butanamine, 1-dodecene, 2-decenal, 3-tetradecene, 2-tetradecene, 1-octadecene, hexadecanoic acid, 10-octadecenoic acid, and heptadecanoic acid.
(TIF)
