# Supplementary figures and images for: Moringa oleifera as an Anti-Cancer Agent against Breast and Colorectal Cancer Cell Lines
Source: PLoS One. 2015 Aug 19;10(8):e0135814. doi: 10.1371/journal.pone.0135814 (PMC4545797; doi:10.1371/journal.pone.0135814)

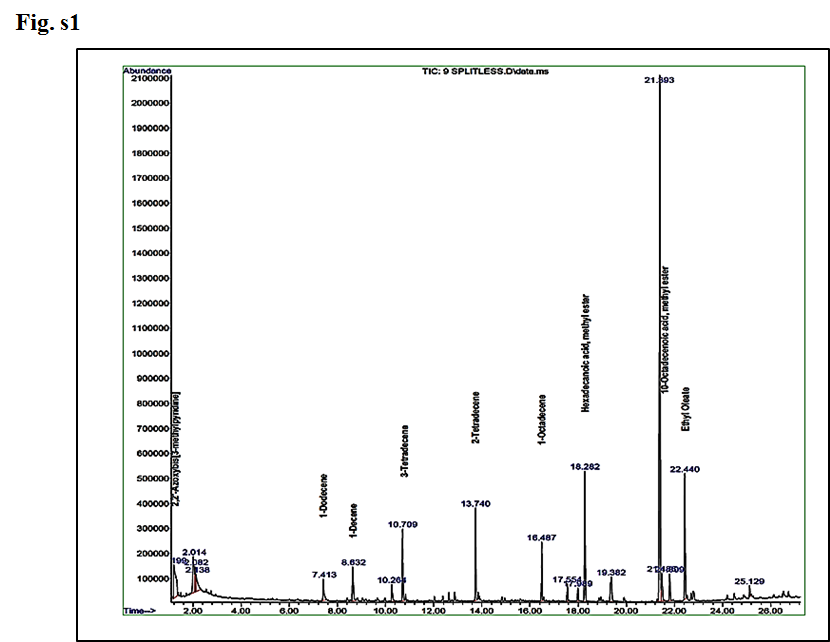

Supplement: S1 Fig — (A) Typical TIC-GC/MS chromatogram of Moringa seed analyzed on GC system equipped with an apolar 5-MS capillary column attached with Mass Detector. It showed the presence of fourteen compounds, these are: 1-butanamine, 1-dodecene, 2-decenal, 3-tetradecene, 2-tetradecene, 1-octadecene, hexadecanoic acid, 10-octadecenoic acid, and heptadecanoic acid. (TIF) [file pone.0135814.s001.tif]
